# Current–Pressure Dynamics Modeling on an Annular Magnetorheological Valve for an Adaptive Rehabilitation Device for Disabled Individuals

**DOI:** 10.3390/mi16020144

**Published:** 2025-01-26

**Authors:** Fitrian Imaduddin, Zaenal Arifin, Ubaidillah, Essam Rabea Ibrahim Mahmoud, Abdulrahman Aljabri

**Affiliations:** 1 Mechanical Engineering Department, Faculty of Engineering, Islamic University of Madinah, Medina 42351, Saudi Arabia; emahoud@iu.edu.sa (E.R.I.M.); aaljabri@iu.edu.sa (A.A.); 2 Mechanical Engineering Program, Faculty of Engineering, Universitas Sebelas Maret, Surakarta 57126, Indonesia; zaenal.arifin22@student.uns.ac.id (Z.A.); ubaidillah_ft@staff.uns.ac.id (U.)

**Keywords:** magnetorheological, MR valve, dynamic modeling, current–pressure relationship, black-box modeling, transfer function, adaptive rehabilitation device, health rehabilitation

## Abstract

The dynamic relationship between current and pressure in magnetorheological (MR) valves is essential for the design of adaptive rehabilitation devices aimed at health rehabilitation for disabled individuals, yet it remains under-explored in existing modeling approaches. Accurately capturing this relationship is vital to predict the pressure drop response to current variations, facilitating the development of effective control systems in such rehabilitation applications. This study employs a linear black-box modeling approach to characterize the current–pressure dynamics of an annular MR valve. Experimental data are used to develop a set of transfer function models, with parameters identified through MATLAB’s system identification tools, utilizing invariant variable regression and the Levenberg–Marquardt (LM) iteration. The modeling yielded a 14th-order transfer function, labeled TF14, which closely aligns with experimental data, achieving a root mean square error of 12.64%. These findings contribute valuable insights into the current–pressure dynamics of MR valves and establish a foundational model for adaptive rehabilitation devices designed for individuals with disabilities.

## 1. Introduction

Magnetorheological (MR) fluid-based technologies have been extensively developed since the discovery of MR fluids by Rabinow et al. in the 1940s [1]. MR fluids exhibit the unique ability to alter their rheological properties in response to changes in the applied magnetic field. This characteristic enables rapid modulation of pressure or force, making MR fluids highly suitable for applications requiring precise and dynamic control. As a result, MR fluids are classified as smart materials, with wide-ranging applications in pressure and force control devices. Among the most prominent MR fluid-based technologies are MR valves [2,3,4,5], MR dampers [6,7,8,9,10,11], and MR brakes, which have been integrated into automotive systems [12,13,14] and medical equipment such as smart prosthetics [15]. Additionally, MR fluid-based devices have found applications in pressure control valves, vibration mitigation systems for buildings, and various household technologies, showcasing their versatility and adaptability across industries.

MR fluid-based devices often rely on MR valves to control the rheological properties of the MR fluid, which directly influences the magnitude of force or pressure generated by these devices. This capability is particularly significant in adaptive rehabilitation devices, where precise control of pressure or force is essential for tailoring therapeutic interventions to individual needs [3,4]. Adjusting the rheological properties of the fluid requires alteration of the magnetic field applied through the MR valve. While there are various methods to achieve this adjustment, electromagnetic control is the most widely used approach [5]. In this method, the magnetic field can be changed simply by modulating the coil’s input current.

There are two types of MR valves based on the structure of the channel: annular and radial MR valves [9,10]. The annular MR valve has an annular channel where the fluid flows in an annular direction, providing a simple and robust design that is easy to manufacture. In contrast, the radial MR valve features a radial channel, making the fluid flow in a radial direction. While radial MR valves may offer improved adaptability to specific configurations, their complex design and manufacturing process often limit their practical applications. Furthermore, the annular valve’s geometry provides more consistent and predictable flow dynamics, which is advantageous for system modeling and control applications. Given these factors, this study focuses on the annular MR valve due to its simpler design, cost-effectiveness, and practical relevance for adaptive rehabilitation systems, where reliability and ease of implementation are key considerations.

The electromagnet’s current-controlled magnetic field permits the control system’s algorithm to automatically adjust the coil’s current supply. Implementing a control system algorithm aids in estimating the correct amount of required input current [6,16,17]. For the control system to figure out the right amount of current, it needs a dynamic model that can show how the MR valve changes over time, especially the relationship between changes in current and changes in pressure over time. The MR valve dynamic model should include a characterization of the pressure drop response to fluctuations in the magnetic field or current. This is crucial for control systems, particularly in rehabilitation devices where precise modulation of force is required to suit individual therapeutic needs.

Existing force–current models often fail to address the transient behavior of MR valves under rapidly changing input currents, focusing instead on steady-state responses or hysteresis effects. This limitation makes them inadequate for real-time control applications, especially in rehabilitation systems where the system must respond dynamically to varying conditions. Additionally, many existing models are either computationally intensive or lack sufficient accuracy, posing challenges for integration into practical control algorithms. Therefore, this study emphasizes the development of a simplified and accurate current–pressure model specifically tailored to address the transient behavior of MR valves, making it highly applicable for real-time control in adaptive rehabilitation systems.

The existing MR damper model consists of two distinct models. The first model is the force–velocity model, which depicts the transient force response resulting from velocity change. A number of researchers have extensively studied the force–velocity model using various techniques. Stanway et al. conducted one of the earliest experiments in 1987 using the Bingham plastic-based model approach to model the force–velocity of MR damper behaviors [18]. According to this study, a straightforward equation can capture changes in maximum force as a result of velocity changes. However, Stanway’s model is lacking in modeling the hysteresis phenomenon in force–velocity behavior. After a decade, Spencer et al. developed the Bouc–Wen model to enhance force hysteresis prediction in 1997 [19]. Spencer’s study offers better model prediction than the Bingham model. Other researchers, such as Lischinsky et al., later developed these two models into a novel parametric model called the LuGre Hysteresis model, which has more flexible and accurate performance than the two previous models [20]. These three early versions of MR dampers are commonly referred to as parametric models. These model types use a physical spring-mass-damper representation method to get close to the MR damper force–velocity behavior.

The second model is the force–current model. This model explains how changes in current input cause a transient force response. A more straightforward method of the force–current model, called the black-box approach, was examined in order to produce a more accurate model in a recent study [21]. This approach uses a numerical and statistical technique to produce a dynamic model from experimental data on the system’s behavior. Ikhtiar et al. developed the first model of MR damper force–velocity behavior by utilizing the Gaussian and Generalized Bell algorithm approaches in 2021 [22]. Their research provides a straightforward way to describe the behavior of MR dampers without requiring a complicated numerical method. This study shows that the Gaussian technique can imitate hysteresis behavior with a maximum relative error of 14%. In 2022, Abdelhamed et al. developed the first model with different black-box method techniques using a nonlinear ARX statistical method [23]. Abdelhamed’s model is significantly more accurate than the Bouc–Wen model, with a root mean square error of only 6.2%.

Nonlinear modeling techniques such as neural networks and nonlinear ARX models have shown great promise in capturing the complex nonlinear dynamics of MR devices. Unfortunately, they come with distinct advantages and disadvantages. Neural networks are highly flexible and capable of modeling hysteresis and other nonlinearities without requiring prior assumptions about the system. However, they are computationally expensive, heavily reliant on large datasets, and often lack interpretability. Nonlinear ARX models, on the other hand, provide a balance between accuracy and interpretability and are easier to integrate into control systems. However, their performance depends on expert knowledge for model structure selection and may be limited when modeling highly complex behaviors. While these advanced techniques are valuable, they often involve trade-offs between complexity, accuracy, and computational demands. Meanwhile, although the force–velocity model has been significantly improved and provides accurate results, this model cannot explain the force change behavior as a result of a rapid change in the amount of input current. The force–current behavior is really crucial since it is the only access to the controller intervention when the MR damper is involved with a control system. The option for modeling this behavior is by using the force–current MR damper model, particularly through the MR valve model. Yet, little progress has been made in developing the force–current model. Therefore, to obtain a representation of the MR valve current–pressure behavior, the pressure drop-current model is the main focus of this study.

In this study, a linear black-box approach is utilized to develop a simplified current–pressure model for MR valves, focusing on computational efficiency and practical applicability in real-time control systems. This is particularly relevant in adaptive rehabilitation devices where scalability is a key factor due to the need for rapid and precise current modulation to adapt to varying user conditions. The transient response modeling of MR valves plays a critical role in enabling such scalability. To achieve this, some assumptions and considerations are taken to preserve the modeling process, focusing on the transient effects of current changes on pressure drop changes. This study’s modeling process focuses on the MR valve part since its contribution to the current–force change behavior is significant. Based on prior studies, the black-box model provides more straightforward and accurate methods for simulating MR damper behavior. Therefore, the black-box method is used for the modeling process in this work, and the output of the model is a linear transfer function model that makes a simple current–pressure model.

## 2. Research Method

In this study, the current–pressure behavior of the MR valve is modeled using a linear black-box model with a transfer-function model as the output model. In this investigation, an annular bypass MR valve was utilized. The experimental testing data of the MR valve were utilized to predict the valve’s behavior according to current–pressure changes. In the experimental testing data, the variations in the amplitude of the current were identified as input data, whereas the changes in pressure drop were identified as output data.

### 2.1. Magnetorheological Valve Design

**Figure 1:**
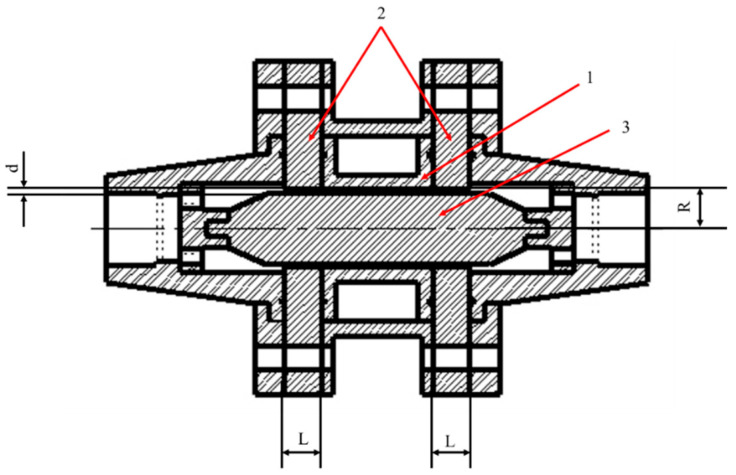
Annular MR valve design.

The type of magnetorheological (MR) valve used in this study is an annular MR valve. The structure of the annular MR valve makes an annular-shaped channel, so the fluid will flow through this channel and be magnetized in this channel. This annular MR valve offers a simpler construction and is easier to fabricate than the radial MR valve, resulting in a significantly lower price. Figure 1 shows the design of the annular MR valve utilized in this study. It consists of three major components: (1) the coil bobbin, (2) the magnetic disk, and (3) the magnetic core. The coil bobbin (1) was fabricated from aluminum 1100 to prevent magnetic flux leakage, while the magnetic disc (2) and the magnetic core (3) were fabricated from AISI 1010 carbon steel grade to provide a strong magnetic flux density. Furthermore, the effective length of the magnetization region (L), the annular channel width (d), and the radius of the annular channel (R) are listed in Table 1. The electromagnetic parts are arranged by 600 turns of the AWS 24 electromagnetic coil and installed in the coil bobbin (1).

In this study, we used the MRC-C1L series MR fluid from CK Materials Lab, which is often used on a linear shock absorber. This fluid contains two primary components: metal particles and a carrier fluid. The metal particles are made of ferromagnetic materials with a diameter of between 1 and 10 m, while the carrier fluid consists of organic or aqueous fluids. This MR fluid shows a rapid response to a change in a magnetic field, with it only requiring 1–20 milliseconds of response time. Table 1 describes the fluid properties of the MRC-C1L MR fluid. In this study, the MR fluid used is an MR fluid with the MRC-C1L series from CK Materials Lab, South Korea, which was applied to a linear suspension system. The basic properties of this MR fluid are described in Table 2.

### 2.2. Magnetic Simulation

There are many things that affect how well an MR valve works, including the design of the annular channel dimensions, the materials used in the valve, the types and numbers of electromagnetic coils, the type of MR fluids, and the number of coils. Thus, the performance of the MR valve and its ability to generate a certain amount of pressure drop must be reviewed because they depend on the magnetic flux acting on the MR fluid [16]. The amount of magnetic flux depends on several criteria, as mentioned before. Hence, with the defined MR valve design, the magnetic flux value in the annular channel was calculated through magnetic simulation.

**Figure 2:**
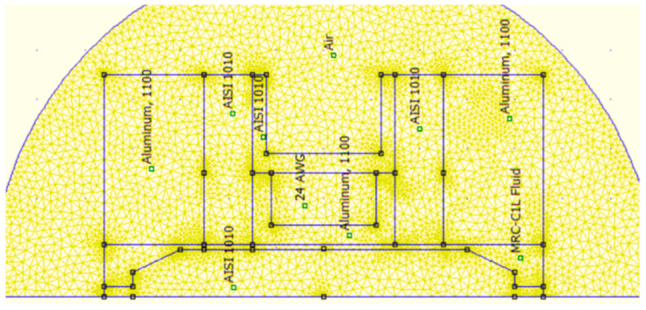
Annular MR valve mesh view using FEMM software 4.2.

**Figure 3:**
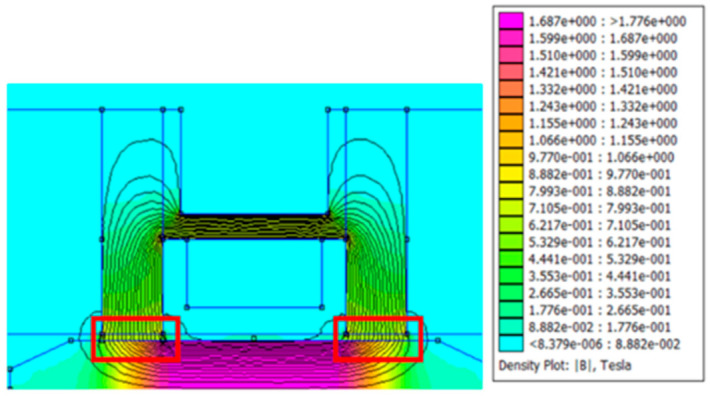
The distribution of the magnetic flux value on the MR valve annular channel.

There is a mesh view and the value of magnetic flux in the magnetization area in Figure 2 and Figure 3. They show an FEMM simulation of an annular MR valve. Based on Figure 3, the magnetic field produced by the MR valve at a current input of 1 A is roughly 0.7 T at the MR fluid gap shown in the red box. As a result, according to Equation (1), it is possible to calculate the pressure drop using the quasi-static fluid behavior approach that Bingham et al. proposed. Equation (1) illustrates how Bingham et al.’s [24] quasi-static fluid behavior approach led to the determination of this pressure drop value:

(1) $$\tau =\tau \left(H\right)sgn\left(\dot{\gamma }\right)+\eta \dot{\gamma }$$

where the total shear stress (τ) is the total sum of the yield stress; τ(H) is the yield stress, which depends on the magnetic flux value; $\dot{\gamma }$ is the shear-strain rate; and η is the dynamic fluid viscosity. The total shear stress in Equation (1) must be converted to a pressure drop by calculating the annular channel’s size. Thus, Equation (2) was used to calculate the annular MR valve performance.

(2) $${∆P}_{total}=\frac{6\eta QL}{\pi d^{3}R}+\frac{c\tau (H)L}{d}$$

The total pressure drops (∆P_total_) is the sum of viscous and yield stress pressure drops. The viscous pressure drop is influenced by the fluid viscosity (η); the flow rate (Q); the magnetization channel length (L); the annular channel inner area (πd^2^); annular channel gap (d); and the annular channel radius (R). In comparison, the magnetic yield pressure drop is influenced by the velocity profile coefficient (c); yield stress due to the magnetic field (τ(H)); magnetization channel length (L); and annular gap (d). To obtain the velocity profile coefficient (c), it must be calculated using Equation (3):

(3) $$c=2.07+\frac{12Q\eta }{12Q\eta +0.8\pi Rd^{2}\tau (H)}$$

## 3. Experimental Setup and Modeling

### 3.1. Experimental Setup

In this study, black-box modeling was used to model the MR valve current–pressure behavior. The black-box technique requires the use of experimental data as the basis for model calculation. The experimental data were collected by examining the pressure drop response to changes in input current. The experimental test process was performed by pumping the MR fluid through the MR valve channel at a steady speed of between 5 and 10 mm/s. As the fluid flows through the annular channel, the input current will rapidly change from the off-state (0 A) to the on-state at the required current magnitude several times. Table 3 summarizes the changes in magnitude variations of currents.

The on–off condition is controlled by using the on/off switch, so the changes in current input form a step signal. The source of current input applied to the MR valve is generated using a 0–30 A DC power supply.

**Figure 4:**
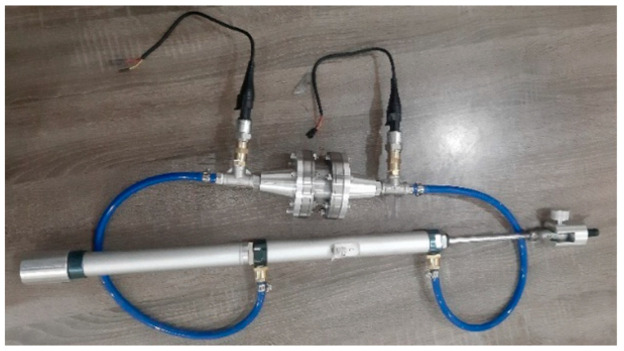
MR valve and double-ended cylinder setup.

**Figure 5:**
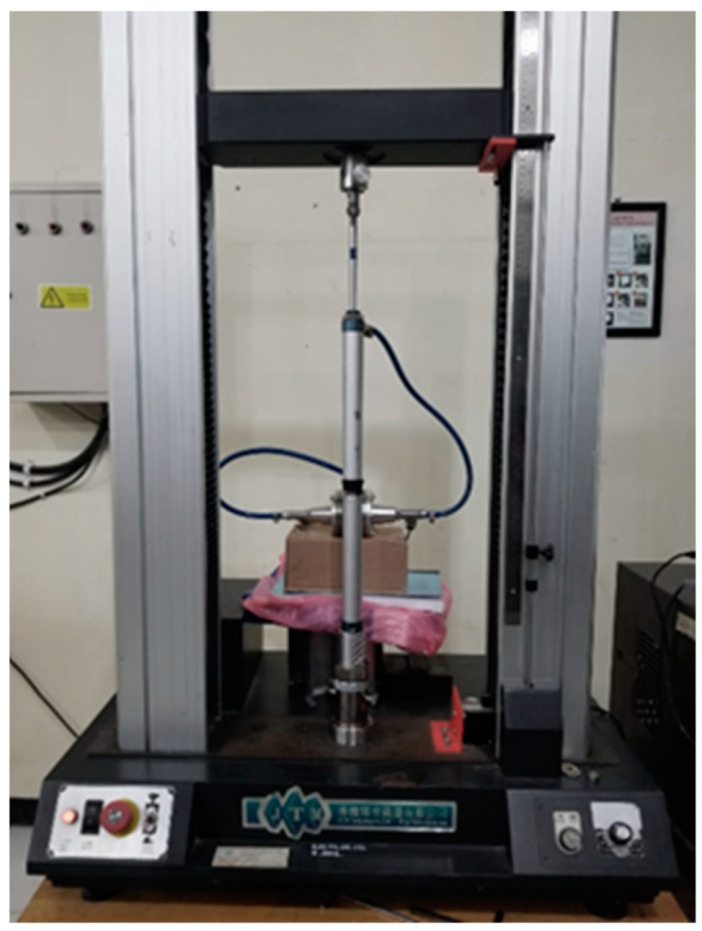
Experimental setup.

A double-ended pneumatic cylinder is utilized to pump the MR fluid to flow through the annular MR valve channel. Figure 4 shows the setup of the MR valve and double-ended cylinder. As illustrated in Figure 5, the double-ended cylinder is fitted on the universal testing machine, so the cylinder piston rod can be pushed at a constant speed.

**Figure 6:**
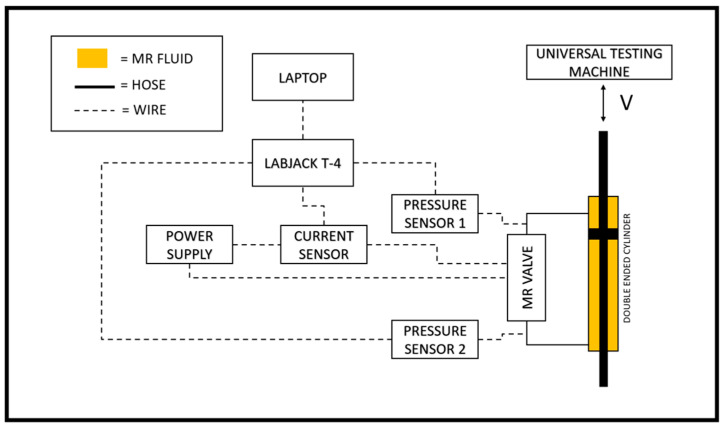
Schematic of the instrumentation setup.

Two sensors are utilized to measure experimental data. The first sensor is a pressure transducer sensor with a measuring range of −12 to 12 bar, while the second sensor is a DC current sensor with a measuring range of 0 to 30 A. These sensors measure the rapid transient response of pressure drop changes and current changes. With a sample rate of 0.002 s, the LABJACK T-4 series data acquisition device (LabJack Corporation, Lakewood, USA) is used to record transient data measurements. These recordings are recorded in CSV format for the purposes of analysis and modeling. Figure 6 shows the schematic and experimental setups, respectively. To ensure measurement validity, the experimental data are measured three times for training data and seventy-two times for testing data. All data measurements contain current–pressure drop behavior. This data series is then modeled using the linear black-box approach.

### 3.2. Linear Black-Box Modeling

Black-box modeling is a statistical method for generating a model from input–output measurement data. These input–output data are the product of experimental testing. Hence, a deep understanding of the system’s principles is not necessary to help this modeling method. The black-box method is helpful for modeling systems that physical analysis cannot reach because of this advantage. However, understanding the system’s basic principles can help in finding the closest convergence, so the trial-and-error step can be reduced.

There are several black-box modeling approaches. In general, there are two approaches to black-box modeling: linear and nonlinear approaches. The linear approach provides a simpler and more robust model, which is ideal for describing a linear system. Yet, some situations involving nonlinear systems can be handled using a linear technique, although a higher-order equation would be required to provide more accurate modeling results. The transfer function and state-space model approaches are examples of the linear black-box approach.

In comparison, the nonlinear technique provides a more accurate model and can be used to describe a nonlinear system. Yet, the nonlinear approach offers a wide variety of methodologies, and the risk of an unstable model outcome is significantly greater than with the linear approach. This method employs numerous nonlinear statistical techniques, including artificial neural networks, nonlinear ARX, ANFIS, and polynomial models.

The general step technique for both linear and nonlinear black-box approaches is the same. The steps for defining a model using the black-box method are as follows:

1. Prepare the experimental data that contain input and output variable relationships. They can be obtained from SISO and MIMO systems, but in the transfer function case, it is recommended to only model the SISO system.
2. Analyze the system and define the types of black-box approaches, linear or nonlinear. In this step, it is important to use the correct method so the best result can be obtained easily without too many trials and errors.
3. Conduct a modeling process from experimental data with a preferred approach to obtain the best model. In many cases, the modeling process is performed several times to achieve the best and most acceptable results.
4. Conduct a validation of the best model performance. Although the best model is achieved, it needs to be validated with the other experimental data in the same systems with different variations to ensure the robustness and sustainability of the best model.

Kostamo et al. conducted research that led to this choice of approach [24,25]. They created a hysteresis MR valve model using a transfer function linear black-box model. The authors produced a six-order transfer function model with an RMS error of 10%. Furthermore, the transfer function model provides a simple equation that is suitable to be applied to numerous control system algorithms. In this study, a linear black-box model approach with a transfer function model approach was used to describe the current–pressure behavior of the MR valve. The selection of high-order transfer function models is guided by the need to accurately capture the dynamic behavior of the system, particularly the hysteresis and nonlinear effects that emerge during current variations. Higher-order models enable a more precise approximation of the system’s response by increasing the number of poles and zeros, which enhances the model’s ability to fit the experimental data. The trial-and-error process for determining the optimal model order was supplemented by the analysis of residuals and goodness-of-fit metrics, ensuring the model complexity remained balanced against computational efficiency.

The transfer function model is one of the linear models that is in mathematical equation form and is often used as a representation of linear time-invariant system behavior. This model is an effective instrument for modeling single-input and single-output (SISO) systems because of its simplicity and robustness. Even so, some linearized nonlinear systems can also be modeled using this method. The model of the transfer function can be expressed as a differential equation through an inverse Laplace transformation. Hence, the physical model of systems can be constructed using the transfer function model. There are two ways to approach the transfer function model: continuous and discrete time approaches. In this work, a continuous strategy is taken to assure the model’s robustness. The general form of the continuous transfer function is shown in Equation (4).

(4) $$TF\left(s\right)={\left.\frac{L[Y(t)]}{L[X(t)]}\right|}_{zero\;initial\;condition}=\frac{Y(s)}{X(s)}$$

where the transfer function (TF(s)) is the ratio between the output (Y(t)) and input (X(t)) variables. The (t) terms represent a function or differential equation in the time domain. Since the input and output variables of the transfer function are in the frequency domain (s), the differential equation must be transformed into the frequency domain using the Laplace transformation. As described in Equation (5), the outcomes of the Laplace transforms Y(s) and X(s) can be defined by the series of poles and zeros ratio.

(5) $$\frac{Y(s)}{X(s)}=\frac{b_{0}s^{m}+b_{1}s^{m-1}+\cdots +b_{m-1}s+b_{m}}{a_{0}s^{n}+a_{1}s^{n-1}+\cdots +a_{n-1}s+b_{n}}$$

where b and a stand for zeros and poles and m and n stand for zeros and poles.

To estimate the parameters of the transfer function model, instrumental variable regression and the Levenberg–Marquardt (LM) optimization technique were utilized. These methods ensure that the coefficients of the poles and zeros are optimized to minimize the error between the model output and the experimental data. The LM algorithm was particularly chosen for its robust performance in nonlinear least-squares problems, which is crucial for achieving convergence in higher-order models. MATLAB 2021a’s System Identification Toolbox facilitated the iterative optimization process, providing tools to fine-tune the parameters and validate the results.

In the first step of this current–pressure behavior modeling, the experimental data are separated into training data and testing data. The training data contain information about input–output data for the entire current magnitude change variation. While the testing data contain some parts of the training data, the training data are utilized to develop a model of transfer function structure. The distinction between each set of transfer function structures is based on the number of zeros and poles and their coefficients. After the entire modeling process is performed, three transfer function structure models with the lowest error rate, less than 30%, will be chosen and tested with test data to ensure their sustainability and robustness. The transfer function model with the highest accuracy in the testing process is selected as the final model.

## 4. Results and Discussion

### 4.1. Experimental Results

**Figure 7:**
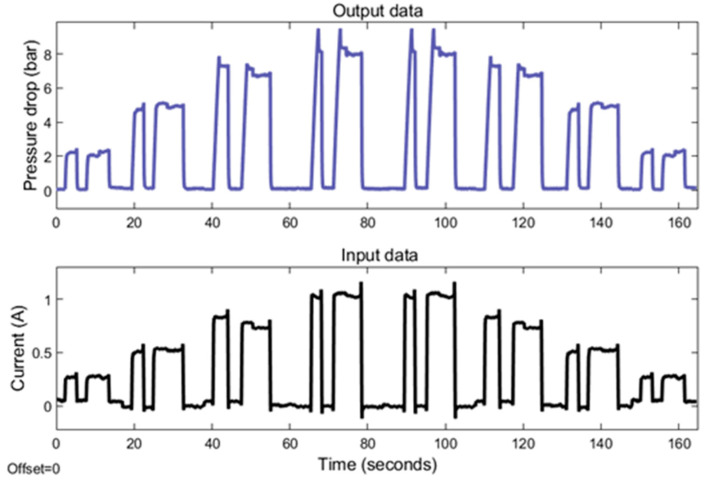
The MR valve experimental data at 5 mm/s velocity.

**Figure 8:**
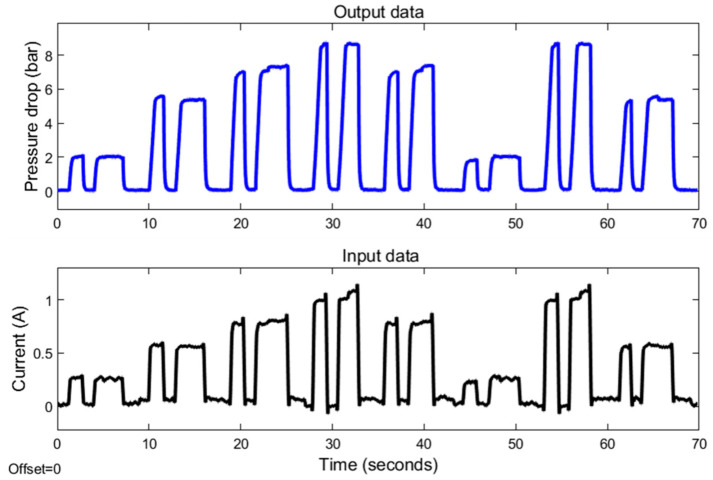
The MR valve experimental data at 10 mm/s velocity.

An annular MR valve was subjected to experimental data testing by utilizing the approach and variation stated in Figure 6 and Table 3. Figure 7 and Figure 8 show the results of experimental tests conducted at constant speeds of 5 and 10 mm/s. The experimental results reveal the transient response of the pressure drop for all changes in the amplitude of the current under off–on scenarios. The results reveal the time required for the pressure to drop and the current to switch from an off state to an on state for each magnitude variation of the current. The pressure drop rising time at 5 mm/s variation for current magnitudes of 0.25, 0.5, 0.75, and 1 A is 299, 572, 1026, and 1164 ms, respectively, while the pressure drop rising times for the 10 mm/s variations are 276, 415, 529, and 605 milliseconds. In contrast, the rising time of all current magnitude changes is towards a constant value. The average current rise time is 39.4 milliseconds.

The phenomenon of rise time describes the most dynamic aspect of the current–pressure behavior of an MR valve. Based on this rise time phenomenon, the pressure drop rise time requires additional time to transition from an off to an on state than the current rise time. The finding also indicates that the duration of the pressure drop at a velocity variation of 10 mm/s rises proportionally as the range of current changes increases. In contrast, the pressure drop increase time at 5 mm/s velocity variation exhibits a nonlinear correlation with the variations in current. Yet, the covariance between the pressure drop rise time and the range of current changes is highly positive. This nonlinear relationship can thus be linearized.

In addition, the experimental testing also shows the difference in pressure drop amplitude as a result of the changes in current input magnitude. At both 5 and 10 mm/s velocities, the value of this amplitude increases as the amount of applied current increases. At a 5 mm/s velocity variation, the pressure drop amplitude values at 0.25, 0.5, 0.75, and 1 A are 2.50, 4.89, 7.19, and 8.5 bar. In comparison, the pressure drop amplitudes for 10 mm/s variation are 2.39, 5.40, 7.40, and 8.84 bar. These growing amplitude values at both velocities indicate a strong positive correlation with the amount of current input. Hence, the relationship between the amplitude of the pressure drop and the size of the current can be linearized. The modest difference in pressure drop amplitude between 5 and 10 mm/s variation, in which the pressure drop amplitude at 10 mm/s has a greater value, is due to the viscous effect of the fluid.

The experimental results for 5 mm/s showed several irregularities. The existence of pressure drop overshoot at the 0.75 and 1 A current magnitudes and the nonlinear relationship between pressure drop and current change rise time indicate these anomalies. These anomalies may be due to the presence of air in the fluid and the hysteresis phenomena at the transition between the pre-yield and post-yield regions.

### 4.2. Modeling Results

The experimental results indicate that the input and output data have a significant positive correlation with the current–pressure behavior. Thus, this behavior can be modeled using a linear approach through the linearization step.

**Figure 9:**
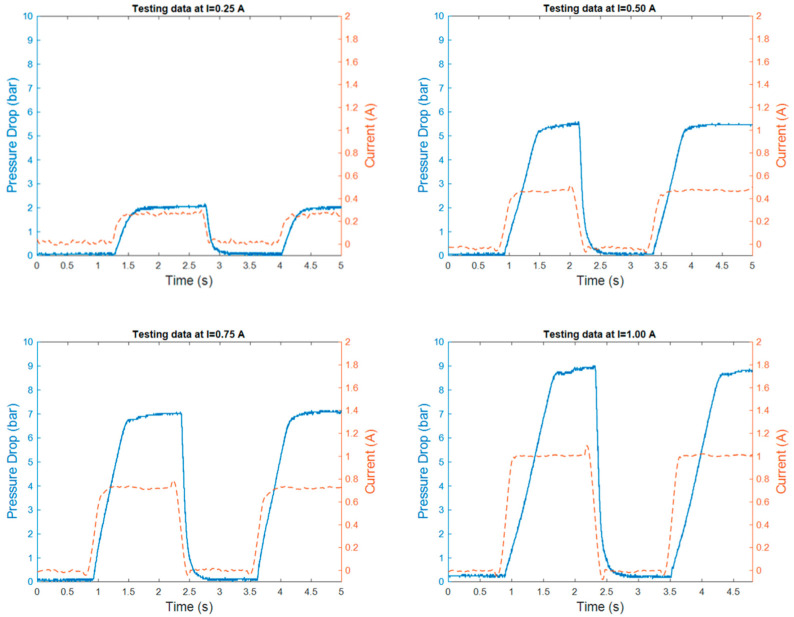
The sample of the MR valve experimental results.

The modeling procedure begins by separating the experimental results into training and testing data. As shown in Figure 7 and Figure 8, the training data are derived from the experimental data with random variety of inputs. Meanwhile, as shown in Figure 9, the testing data are gathered through various experimental tests with specific current inputs. The training and testing data are imported into the system identification toolbox. Using a number of transfer function model structures, this program models all training data. Table 4 and Table 5 list the series of transfer function structures and their performance. The model’s performance is computed using root mean squared error (RMSE). The RMSE equation is shown in Equation (6), where y is the output of experimental data and $\hat{y}$ is the output of the model.

(6) $$RMSE=(\frac{\left|y-\hat{y}\right|}{\left|y-mean\left(y\right)\right|})$$

The series of transfer function structures modeled at 5 and 10 mm/s have varied coefficients despite having identical poles and zeros. Thus, the performance of transfer function structures that have the same number of poles and zeros may vary. As shown in Table 4 and Table 5, using transfer function structures with higher-order structures is a good way to accurately model the dynamics of how the current and pressure behave. Yet, in the 5 mm/s velocity variation results, some of the transfer function structures have poor performance, with an RSME value of more than 30%. In comparison, the performance of the transfer function structures from 10 mm/s modeling is better, with certain model structures having an error of less than 30%. The poor performance of the 5 mm/s model is due to the significant nonlinear relationship between the pressure drop and the rise time of the current change. Hence, linearization procedures are less effective in generating accurate models.

**Figure 10:**
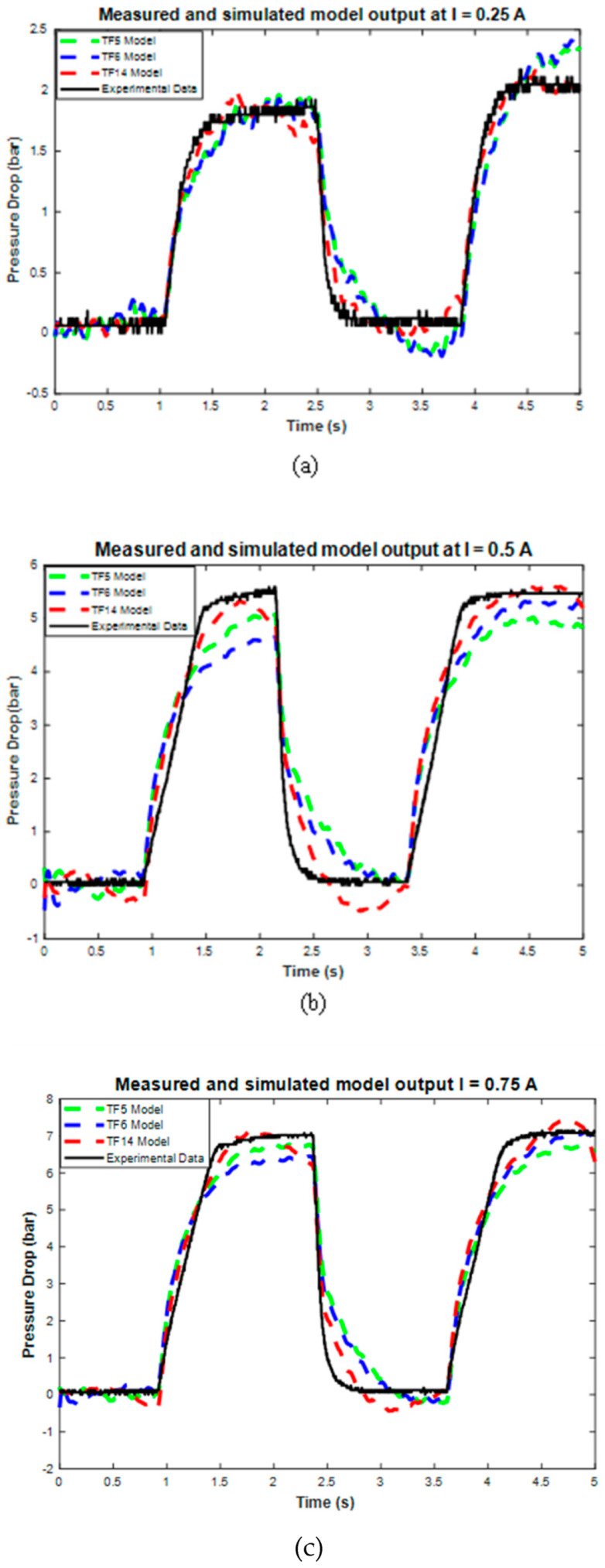

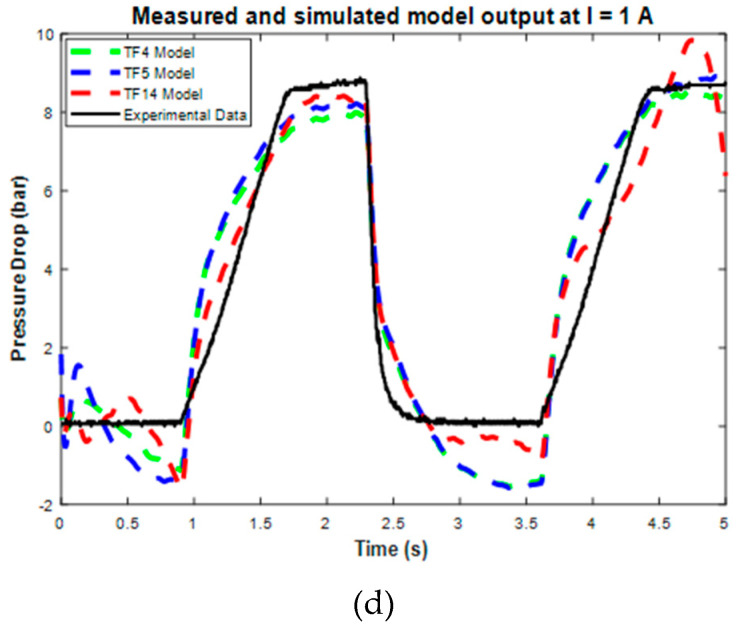
The performance of the top three transfer function model structures on the sample of all testing data current variations.

Data modeling at 10 mm/s produces the three best transfer function model structures, as shown in the modeling results. These top three model structures are TF5, TF6, and TF14, which have RMSE values of 28.36%, 28.41%, and 28.48%. The best three models are then tested using 72 testing data points. Figure 10 and Table 6 show the sample and average performance results of the three best transfer function models with testing data.

Furthermore, the validation results also show the value of the RMSE error for each model’s performance, as shown in Table 6. The RMSE results show that TF5 has the largest RMSE with a 20.21% error value, while TF14 has the smallest RMSE with a 12.64% error value. Therefore, based on these RMSE and graphical results, TF14 was selected as the final transfer function model because it has the best accuracy compared to TF5 and TF6. However, TF14 still has some weaknesses, such as the excessive approach in the on-off conditions, which caused an undershoot phenomenon, and the excessiveness in the steady state error. Despite these weaknesses, the TF14 model still has good accuracy and can be used to model current–pressure behavior with good performance. The complete TF14 model structures are shown in Equation (7), and the coefficients of each pole and zero are listed in Table 7.

(7) $$TF14=\frac{as^{13}+bs^{12}+\cdots +ks^{3}+ls^{2}+ms+n}{As^{14}+Bs^{13}+\cdots +Ls^{3}+Ms^{2}+Ns+O}$$

The performance of the 14th-order transfer function model (TF14), as evidenced by its low RMSE of 12.64%, demonstrates its ability to accurately capture the dynamic current–pressure behavior of the MR valve under rapid input changes. This accuracy, combined with the computational simplicity of the linear black-box approach, highlights the model’s scalability for applications requiring rapid current modulation. Such scalability is particularly critical in adaptive rehabilitation devices, where real-time responsiveness is essential for delivering personalized therapeutic interventions [26,27,28].

## 5. Conclusions

In this study, black-box dynamic modeling was performed to characterize the current–pressure behavior of an annular MR valve. The modeling utilized a linear transfer function approach, with system identification carried out using MATLAB. Experimental input–output data, comprising current changes as input and pressure drop changes as output, were obtained through testing of the annular MR valve.

The data were modeled and analyzed using invariant variable regression (IV) and Levenberg–Marquardt (LM) iterations to derive the transfer function models. The results revealed that the current–pressure dynamics could be effectively represented using higher-order transfer function structures. Among them, the 14th-order transfer function (TF14) was selected as the final model, achieving a root mean square error (RMSE) of 12.64% in validation tests.

While the TF14 model demonstrates strong predictive accuracy, there remains significant potential for improvement, either by refining the transfer function structure or exploring alternative modern modeling techniques. Importantly, this study confirms that current modulation significantly influences the pressure drop behavior in MR valves. This finding underscores the necessity of incorporating such dynamic models into the control systems of MR-based devices, particularly for adaptive rehabilitation devices. By enabling precise control of pressure or force, these models can play a crucial role in optimizing therapeutic outcomes for individuals with disabilities.

This study confirms the scalability of the proposed model for real-time control applications, particularly in adaptive rehabilitation devices. By enabling precise control of pressure or force, the model supports rapid current modulation, ensuring its applicability to a wide range of therapeutic settings.

The broader impact of this research lies in its potential to inspire the development of more adaptive systems in medical technology, particularly those requiring real-time control and customization, such as smart prosthetics and assistive devices for disabled individuals. The findings presented in this study offer valuable insights for advancing medical technology systems that demand precise modulation of force and pressure.

Future research could explore the integration of nonlinear methods, such as artificial neural networks or adaptive neuro-fuzzy inference systems, into the current framework to enhance the model’s ability to capture complex dynamics. Additionally, hybrid modeling approaches that combine the strengths of linear and nonlinear techniques could provide a pathway to achieving even greater accuracy and flexibility. Investigating the real-world implementation of the proposed model in active control systems and assessing its performance under varying operational conditions are also critical future directions.

## Figures and Tables

**Table 1:** Annular channel dimensions.

Components	Dimensions (mm)
Magnetization effective length (L)	10
Annular channel width	1
Annular channel radius	19.5

**Table 2:** MRC-C1L fluid properties.

Appearance	Dark Grey Liquid
Density (g/m^3^)	2.75–2.95
Viscosity (Pa s @40 °C)	0.106 ± 0.02
Shear Stress (kPa @570 mT)	68.0 ± 7
Operating Temperature (°C)	−40–140
Flash Point (°C)	>140
Solid Wight Percentage (wt%)	77–80
Sedimentation Stability (vol%/30 days)	4.00

**Table 3:** The current changes and variations.

Variation	Condition	Current (A)
1	ON–OFF	0.25
2		0.5
3		0.75
4		1.00

**Table 4:** The result of transfer function modeling in each structure at 5 mm/s velocity.

Transfer Function	Number of Orders		RMSE (%)
	Poles	Zeros	
1	1	0	30.445
2	2	1	30.29
3	3	2	30.18
4	4	3	31.035
5	5	4	30.86
6	6	5	30.85
7	7	6	30.73
8	8	7	30.91
9	9	8	31.47
10	10	9	30.75
11	11	10	31.405
12	12	11	41.375
13	13	12	39.46
14	14	13	82.761
15	15	14	65.255

**Table 5:** The result of transfer function modeling in each structure at 10 mm/s velocity.

Transfer Function	Number of Orders		RMSE (%)
	Poles	Zeros	
1	1	0	28.53
2	2	1	29.12
3	3	2	28.52
4	4	3	28.88
5	5	4	28.365
6	6	5	28.41
7	7	6	28.79
8	8	7	28.77
9	9	8	64.835
10	10	9	76.7145
11	11	10	36.43
12	12	11	33.08
13	13	12	30.555
14	14	13	28.48
15	15	14	145.99

**Table 6:** The best three transfer function models for validation.

Transfer Function	RMSE of Validation Results (%)
TF5	20.21
TF6	19.77
TF14	12.64

**Table 7:** The TF14 poles and zeros coefficient.

Zeros		Poles	
Notation	Coefficient	Notation	Coefficient
A	−41.8	A	1
B	5316.1	B	62.2
C	−127,314.3	C	5691.3
D	21,142,462	D	247,801.3
E	143,409,991.3	E	6,227,203.6
F	365,489,941	F	22,415,111.8
G	730,969,627	G	60,951,145
H	1,111,865,214	H	103,813,779.8
I	979,829,670.8	I	153,202,135.3
J	702,601,785.7	J	128,318,971.5
K	205,538,612.5	K	87,979,344.5
L	77,012,779	L	26,846,224.5
M	2,555,603.7	M	9,998,137.5
N	776,563.5	N	292,442
		0	103,749.2

## Data Availability

The original contributions presented in this study are included in the article. Further inquiries can be directed to the corresponding author.
